# Final-year student nurses’ experiences of caring for patients

**DOI:** 10.4102/curationis.v43i1.2033

**Published:** 2020-03-02

**Authors:** Sewela C. Kobe, Charlene Downing, Marie Poggenpoel

**Affiliations:** 1 Department of Nursing, University of Johannesburg, Johannesburg, South Africa

**Keywords:** nursing, nursing students, experiences, qualitative research, caring, patients

## Abstract

**Background:**

Shortage of nurses in South African hospitals has affected the nurse–patient ratio, thus prompting nurses to be focussed on completing nursing-related duties with less or no caring for the patient. Caring involves having a therapeutic relationship with the patients, and it can be challenging and demanding for final-year student nurses who are still novices in the nursing profession.

**Objectives:**

To explore and describe the experiences of caring for patients amongst final-year student nurses in order to develop and provide recommendations to facilitate caring.

**Methods:**

A qualitative, descriptive and contextual design was used. Data collection was done through eight in-depth individual interviews. Giorgi’s five-step method of data analysis was used, along with an independent coder. Measures to ensure trustworthiness and ethical principles were applied throughout the research.

**Results:**

Four themes with 12 subthemes emerged from the data: therapeutic relationship with patients as an integral part of caring, teamwork – team spirit makes caring easy, continuous caring that promotes quality and safe nursing, as well as satisfaction amongst staff and patients, and various barriers that contributed to lack of caring in the unit.

**Conclusion:**

The majority of student nurses had positive experiences of caring, which included therapeutic relationships between nurses and the patients, teamwork and team spirit that fostered safe and quality nursing care, rendered effortlessly. Barriers to caring were also highlighted as negative experiences.

## Introduction

Final-year nursing students are expected to care for patients during the clinical allocation of their training. Caring is one of the attributes student nurses should exhibit during training, and it is a quality that should continue after training to enable them to render quality nursing care. Final-year student nurses are destined to become the professional nurses of tomorrow, and they will be expected to function independently to facilitate patients’ health.

The caring attitude and responsibility, which they would need as professional nurses, need to be developed whilst they are still student nurses. Therefore, careful attention to the quality of students’ clinical learning experiences about caring is needed to prepare them to be effective and caring practitioners in the future (Watts & Davis 2014:1153). Adamson and Dewar’s (2014:161) study showed that educators need to ensure that curriculum content actively prepares the final-year student nurses for caring, in order for them to learn and develop values of caring. A study by Baillie et al. (2015:679) noted that caring is an essential part of the final-year student nurses’ role, and that they must be prepared for caring during their years of training. The knowledge and skills that have been imparted to them about caring will assist them to improve the quality of their caring attitudes towards patients. Rodrigues et al. (2016:5) added that there is a need for the development of caring attitudes amongst final-year student nurses in their caring activities with patients.

The purpose of this research was to develop an understanding of final-year student nurses’ experiences of caring for patients and to provide recommendations to facilitate caring by final-year student nurses for patients. The study explored and described final-year student nurses’ lived experiences of caring for their patients.

## Background and literature review

Shortage of nurses in South African hospitals has affected the nurse–patient ratio, thus prompting nurses to be focussed on completing nursing-related duties with less or no caring for the patient. Caring involves having a therapeutic relationship with the patient where nurses interact with patients when performing their nursing duty. Patients appreciate a caring nurse, and they feel the essence of caring from nurses. When there is a caring environment in the unit, patients feel welcomed, respected and their compliance to treatment improves, thus reducing the number of readmissions. Final-year student nurses are expected to care for patients as part of training in order to become qualified registered nurses who are safe practitioners and beneficial to the society. In order to render quality nursing care, caring is an attribute, which final-year student nurses need to learn and acquire whilst they are still in training.

In nursing, caring also encompasses Ubuntu, translated as ‘human kindness to others’, and patient satisfaction (Downing & Hastings-Tolsma 2016:215; Jooste 2010:7). Nurses are in a unique position to engage in caring to promote health and improve the well-being of ailing patients. Caring is the central practice of nursing, and it exists in every society (Jooste 2010:7). It is a shared experience in which nurses and patients interact to facilitate the overall health of the patient (Watson 2005:34). According to a South African historical perspective, caring involves the application of Ubuntu, which promotes the standard of moral behaviour amongst people. It embraces tolerance and fairness and includes reaching out and relating to others in a meaningful and trusting way (Downing & Hastings-Tolsma 2016:215; Jooste 2010:7).

Nurses are expected to possess knowledge and skills to contextualise caring in order to meet the unique needs of individuals (Loke et al. 2015:421–429). South African public hospitals are dedicated to provide compassionate and quality nursing care for patients, but the rise in medico-legal and negligence cases has raised a concern (Nt’sekhe 2018:np). The National Department of Health has become one of the distressed departments because of the high numbers of complaints, allegations and many challenges regarding caring of patients in public hospitals (Pelompe 2018:np). Jooste (2010:24) alludes that compassion, commitment, confidence and competence are the attributes of caring that final-year student nurses should develop during their training. It is recommended that final-year student nurses should be exposed to clinical areas to gain caring experiences and attributes (King-Okoye & Arbber 2014:448). This view is also supported by Yuh-Shiow et al. (2016:3317), who added that caring behaviour amongst final-year student nurses in a clinical setting is essential and can be developed by being exposed to a caring environment.

Caring by novice student nurses can be challenging and demanding, as it involves an interconnectedness of body, mind, emotions, spirit, and social and cultural relationships between the student nurses and the patients (Temane et al. 2016:2). Yuh-Shiow et al. (2016:3317) stated that the most crucial caring behaviour is ‘knowing the patient’, whilst the least important one is ‘advocating.’ This includes respecting the patients’ and their family’s best interests, and it has been found to be difficult for final-year student nurses to practise.

## Definition of key concepts

### Experience

Experience refers to the process of doing things, seeing things and having things happen to you (*Merriam-Webster* 2017). It also refers to an event or occurrence, which leaves an impression on someone (*Oxford Dictionary* 2017). Experience means observing, encountering or undergoing of things generally as they occur over a course of time (*Free Dictionary* 2017). In this study, experience means final-year student nurses’ observing, encountering and undergoing the caring of patients.

### Caring

Caring is defined as a feeling and exhibition of concern and empathy for others, and showing or having compassion (Collins 2014). It is also referred to as displaying kindness and concern for others, characterised by love, compassion, support and involvement (Jooste 2010:6). In this study, caring means the ability of final-year student nurses to render patient-centred care based on the interaction between a nurse and a patient, which promotes the nurse–patient relationship and increases patient satisfaction.

### Student nurse

A student nurse is a person undergoing education and training in nursing, who has complied with the prescribed conditions and who has furnished the prescribed particulars for a training programme at a nursing education institution. The person must be registered with the South African Nursing Council as a learner nurse (*Nursing Act* No. 33 of 2005). In this study, a student nurse refers to a person who is in his or her final year of study as a nurse at an accredited nursing college and is enrolled as a student nurse under Regulation 425 (SANC Regulation 425 of 1985).

### Patient

A patient refers to a holistic being and is regarded as someone consisting of physical, spiritual, emotional intellectual and social aspects (Awalkhan 2016:98). The patient is a person who is receiving nursing care or treatment in a hospital or has previously received care, whose health needs must be met and health status must be maintained by the nurse (SANC Regulation 387:1). In this study, a patient means a person to whom final-year nursing students are providing patient-centred care in order to promote and restore health.

## Methods and design

In this study, a qualitative, descriptive design was used to describe and understand final-year student nurses’ experience of caring for patients from the student nurses’ point of view.

### Setting

The study was conducted in a nursing college in the Gauteng province of South Africa. Student nurses who were doing their final (fourth) year of study and who had completed 80% of their coursework participated in this research. The participants were rich informants of the context of the study as they were nearing the completion of their training, with a maximum of only 20% of their course work left for the final year of study. Most of them were allocated to a clinical area, namely, the primary healthcare centres and the different units in the hospital.

### Population and sampling

The target population for the study included 122 student nurses registered at a nursing college in Gauteng. Student nurses in their fourth year of study, having completed 80% of their required course work and willing to participate were the inclusion criteria. The purposive sampling method was used for data collection. A total of eight student nurses participated in the interviews until data saturation was reached.

### Data collection

In this study, multiple methods of data collection were used to reach the point of data saturation. This included in-depth individual interviews, observations and field notes. During the interview, the researcher asked a central question to initiate the interview: *how is ‘caring’ in your nursing practice?* The primary mode of interview involved open-ended questions. The major aim of the interview was to explore the participants’ responses to the questions. Each interview lasted for 45–60 min. Subsequent questions were determined by the final-year student nurses’ responses to the central question. The researcher probed for more details until the experience was thoroughly described. Communication techniques that were utilised during interviews included clarification, reflection, probing and summarising (Mack et al. 2011:31).

### Data analysis

Giorgi’s (2013:243–273) five-step method of data analysis was used, which entailed making sense of the whole meaning, discrimination of the meaning of each unit whilst focussing on the phenomenon being researched, transformation of the subjects’ expressions by going through the meaning of the units and reflecting on them to come up with the essence of participants’ experiences, conducting a synthesis of transformed meaning of the units into a consistent statement of the structure of the participants’ experiences and doing the final synthesis where all statements regarding each participant’s experiences were synthesised into one consistent statement of the structure of experiences that described and captured the essence of the experiences of caring. An independent coder who was experienced in qualitative research was involved to reduce the risk of biased decision and personal interpretation through collaboration. The independent coder analysed the transcription of the interviews and compared it with the subset of the researcher’s coding decision for coding consistency. Consensus discussion between the independent coder and the researcher was conducted.

### Ethical considerations

Permission to conduct the study was obtained from the Research Ethics Committee of the Faculty of Health Sciences at the University of Johannesburg, reference number: REC-01-82-2017 as stipulated in the *National Health Act* No. 61 of 2003. The principles of autonomy, beneficence, non-maleficence and justice were also applied (Dhai & McQuoid-Mason 2011:38). Autonomy: the participants were given an explanation and information letter about the proposed study, which included information about their right to voluntary participation, right to withdraw without penalty, a request to sign the informed consent after reading and understanding the letter, request to record interview, as well as their reassurance to confidentiality, anonymity and privacy. Beneficence and non-maleficence: there were no direct benefits for participating in the study, but recommendations were developed and provided to facilitate caring amongst final-year student nurses, to ensure that no harm was experienced by the participants during the study. Justice: the researcher adhered to the principle by ensuring that there was fair recruitment in the process of selection of the participants.

## Results

The results were informed by the participants and were divided into the following sections: socio-economic profiling and themes and subthemes.

### Socio-demographic profile

The research study comprised eight final-year student nurses (fourth year) who had completed 80% of their module work. There were three male and five female participants, with ages ranging from 22 to 47 years. The average age of the participants was 29 years. All participants were Africans, and their home languages were Zulu, Tswana, Venda, Tsonga and Pedi. Six were single, and two were married. Only three participants had children. During the data collection process, all participants were assigned either to hospitals, primary healthcare institutions or clinical institutions or settings.

Themes and subthemes based on the experiences of final-year student nurses of caring for patients are described in Table 1.

**TABLE 1:** Summary of themes and subthemes based on the experiences of final-year student nurses of caring for patients.

No.	Themes	Subthemes
Theme 1	Final-year student nurses experienced a therapeutic relationship with patients as an integral part of caring.	Experienced respecting of patients as part of a therapeutic relationship Experienced showing empathy towards the patients as part of a therapeutic relationship Experienced open communication towards the patients as part of a therapeutic relationship Experienced building trust as part of a therapeutic relationship Experienced loving kindness as part of a therapeutic relationship Experienced involving family members in caring for the patients as being important
Theme 2	Final-year student nurses experienced teamwork – team spirit makes caring easy.	Experienced open communication amongst nurses as part of teamwork that promoted caring
Theme 3	Final-year student nurses experienced continuous caring, which promotes quality and safe nursing, as well as satisfaction amongst the staff and the patients.	Experienced caring, which prompted quality and safety in nursing Experienced that nurses felt appreciated and motivated
Theme 4	Final-year student nurses experienced various barriers that contribute to a lack of caring.	Experienced that caring for an overload of patients was tiring for nurses Experienced negative attitudes that affected nurses’ caring for the patients Experienced caring in which focus was on the routine task and not on interacting with the patients

#### Theme 1. Final-year student nurses experienced a therapeutic relationship with patients as an integral part of caring

From the interviews conducted, it became clear that a therapeutic relationship with the patients was an essential part of caring. Therapeutic relationships between the patients and the nurses created a caring atmosphere and let the patients know that nurses understood how they felt. Participants experienced that the therapeutic relationship enhanced caring, which patients could notice and be grateful for. The participants identified respecting patients, nurses showing empathy towards the patients, open communication with the patients, building trust between the nurses and the patients, loving kindness and involving family members in caring for the patients as part of a therapeutic relationship. The following participant quotes support this theme:

‘Patients being easy to care also mean that, nurses can develop a relationship with the patient. I think that a relationship with the patient enhances caring; patients can notice your care and be thankful.’ (Participant 4, aged 29 years, male)

‘We need to care for the patient to make them better, patients come to the hospital to get better. If we don’t get time to sit down and talk to them about their problems, and explore the other part of the patient’s life, then we won’t be able to manage the patient’s illnesses.’ (Participant 6, aged 23 years, female)

‘There are little things that nurses can do, that can make a great difference to the patients.’ (Participant 3, aged 25 years, female)

A therapeutic relationship is essential for the nurses in creating a caring environment, as it promotes understanding and helps to generate a beneficial relationship between the nurses and the patients (Geyer et al. 2016:278). Feo et al. (2017:54) asserted that establishing a positive and trusting therapeutic relationship with the patient has been long recognised as a vital and effective part of caring. Patients need communication and information to reduce their uncertainty and to understand their situation whilst in the hospital. Brown, Scott and Rossiter (2016:54) emphasised that when there is a poor therapeutic relationship between the nurses and the patients, the quality of caring becomes negatively affected. A therapeutic relationship can be utilised for providing care and compassion, and it allows nurses to understand how care should be delivered to the patients (Percy & Richardson 2018:200).

Respect for human dignity is an essential part of a caring nurse. Participants in this study reported the need for patients to be treated with respect and dignity. The main features of delivering patient-centred care are through compassion, dignity and respect. This is supported by the following quote:

‘I would say caring and respect go together, that patient would not have listened if I was not respectful. Before we can care for patients we need to respect them. Some patients will come to the ward very dirty, we need to care and respect them. Respecting the patients also creates a trusting relationship.’ (Participant 7, aged 24 years, female)

Hinkler and Cheever (2017:26) stated that respect goes beyond accepting the impression or attitude that people have a right to independent decision-making. Nurses, in their professional relationships, need to practise with compassion and respect for dignity, worth, privacy and uniqueness of every individual, unrestricted by considerations of social or economic status, personal attributes or the nature of the health problems.

#### Theme 2. Final-year student nurses experienced teamwork: Team spirit made caring easy

From the interviews conducted, it became clear that teamwork and team spirit were crucial in caring for the patients. Teamwork and team spirit enabled nurses to work together effectively and efficiently to meet the objectives of the unit and to promote quality care. Participants reported that teamwork and team spirit made nurses work together in harmony, and that they were able to help each other in the caring process.

Participants identified open communication amongst nurses as part of teamwork that promotes caring, which includes discussion amongst the staff members about the problems in the unit and giving each other feedback on various ways of promoting teamwork and team spirit. Nursing was continuous and therefore was caring. Effective communication amongst nurses promoted continuation of caring, and this could be noticed during the handover between the different shifts. When the handover was done effectively, there was a continuation of caring from one team to another. Participants also reported that communication took place by sharing information amongst nurses. The sharing of information included discussion on individual patients and how to treat certain conditions. Participants stated:

‘In my whole training I have learnt that nursing is continuous and so is caring. Caring becomes effective when there is communication among nurses. For example, during handover, we hand over to the other shift so that caring can continue even on a different shift….’ (Participant 4, aged 29 years, male)

‘Nurses need to share knowledge and information to promote caring. The information we share as nurses include managing individual patients and their condition.’ (Participant 5, aged 22 years, male)

McEwan et al. (2017:24) defined teamwork as the range of interactive and interdependent behavioural processes amongst team members that transmit team inputs into outcomes. Teamwork includes continuous communication between team members in the unit. Thakur et al. (2016:52) mentioned that effective communication amongst nurses is one of the bases of professional nursing practice and the art of caring comprehensively and holistically for patients. Bello (2017:11) asserted that effective communication amongst team members remains a key factor in the improvement of interpersonal relationships and subsequently the improvement of patients’ care and the quality of patients’ recovery.

Participants experienced that report and feedback amongst nurses was a way of promoting open communication in the unit. Nurses should give each other feedback regarding the conditions of the patient to encourage the continuity of care. They also reported that nurses need to acknowledge feedback from the patient so that they can improve the quality of service. The following quote supported this category:

‘Nurses’ communications in the unit should include giving each other feedback about the patients’ conditions and also receiving feedback from the patients.’ (Participant 3, aged 25 years, female)

Meyer et al. (2009:170) stated that giving each other feedback in the nursing unit promotes an effective two-way communication. The multidisciplinary team can discuss the patients’ progress and/or plan therapy, and the information can be obtained from the daily ward reports when feedback is received. Kodama and Fukahori (2016:217) stated that nurse managers need to give constructive feedback to nurses to increase motivation and inspiration. When there is a problem and the tension is high in the unit, nurse managers need to consider the timing, contemplate when to communicate and give productive feedback about the identified problems and solutions to staff members.

#### Theme 3. Final-year student nurses experienced continuous caring, which promoted quality and safe nursing, as well as satisfaction amongst the staff and the patients

From the data collected, the participants reported continuous caring, which promoted quality and safe nursing, as well as satisfaction amongst the staff and the patients. When there was continuous caring in the nursing unit, patients’ compliance with treatment improved, and patient complaints became less, thus reducing the number of readmissions. Participants also reported that quality care and safe nursing enable nurses to take responsibility and feel proud about executing their nursing duties. The benefits of continuously caring for patients were that patients recovered sooner whilst taking responsibility for their own health. Participants identified caring, which promoted quality and safety of nursing; nurses felt appreciated and motivated as part of continuous care in the nursing unit. Caring meant that patients were assisted on time, which reduced complications and litigations. Nurses took responsibility for their actions and proudly executed their nursing duties. Participants also reported that caring in the unit made patients recover sooner and take responsibility for their own health. Participants explained:

‘When there is caring in the nursing unit, you won’t afford seeing some things not done to your patients, e.g., wound dressing, and this will reduce complaints in the unit, there won’t be disagreements where you find patients complaining about nurses.’ (Participant 7, aged 24 years, female)

‘Yes mam, my caring has grown from being just the scope of practice to something that comes from within. I would say I have become the image of my profession.’ (Participant 8, aged 26 years, male)

Nikfam, Pourghane and Ebadi (2017:6) asserted that continuous caring in the nursing unit is the key to patient satisfaction, cooperation and recovery, thus improving quality of care provided by the nurses. Sarpong et al. (2017:4) conveyed that quality care can be improved by allowing patients and families to participate during the ward rounds and decisions, promoting transparency in decision-making. It was shown that such patients’ and family’s satisfaction improved and there were confidence and trust amongst the nurses. Ultimately, the patients and families recommended the hospital to others.

The participants identified that when the patients complied better with treatment and fully understood their conditions, patient complaints decreased, the number of readmissions decreased and medical risks decreased, as part of caring that promoted quality and safe nursing.

Participants experienced that continuous caring in the nursing unit decreased patient complaints, which prompted nurses to care for the patients without complaints and resentment. Participants also reported that when nurses care for their patients, they need to open the channels of communication so that patients do not communicate to them through a suggestion box or a complaints box, but instead they express themselves if there are problems. This category was supported by the following quotes:

‘When nurses give patients explanations, they understand the situation and that reduces the complaints in the nursing unit because they understand what is going on.’ (Participant 4, aged 29 years, male)

‘We need to open channels of communication so that patient must not communicate to us through the suggestion or complaints box.’ (Participant 5, aged 22 years, male)

Norouzinia et al. (2016:65) alluded that good communication in the nursing unit is a vital element in providing high-quality nursing care, leading to patient satisfaction and health promotion. Increased patient satisfaction, acceptance, compliance and cooperation with the nursing staff improve the physiological and functional status of the patient and thus lead to fewer complaints and dissatisfaction in the nursing unit. Aiken et al. (2017:6) in their study on patient satisfaction found that patients expressed a high level of confidence and trust in nurses, and their satisfaction with hospital care was high. Ensuring an adequate number of nurses at the hospital and improved hospital clinical care environments were strategies used to improve patient satisfaction with caring.

#### Theme 4. Final-year student nurses experienced various barriers that contribute to a lack of caring

From the interviews conducted, participants reported various barriers that contributed to a lack of caring in the unit. These barriers affected caring in the nursing unit by reducing communication in the unit, whereby patients were scared to inform the nurses about their concerns. Patients were also afraid to ask questions about their health and treatment and therefore could not make informed decisions. Participants reported that barriers made patients feel neglected and the quality of nursing became compromised. Different barriers to caring had been noted as an overload of patients, negative attitudes of nurses towards the patients, routine tasks-orientated caring and lack of interaction with the patients. Participants explained:

‘I was working in a surgical ward and there was a patient on a vacuum drainage, which was blocked for the weekend. When I asked her, “why you didn’t ask for help”, she said she feared the nurses who were working over the weekend.’ (Participant 7, aged 24 years, female)

‘Nurses have built a barrier between them and the patients, and patients have accepted nurses as being like that. Nurses need to involve patients in their treatment, but patients fear nurses. We need to come with the solution to remove this fear of nurses.’ (Participant 3, aged 25 years, female)

Woith et al. (2017:217) noted that patients in their study reported an experience of incivility from nurses; they described nurses as not taking their health concerns seriously. Patients did not believe that healthcare providers listened to them. Stalpers et al. (2017:47) alluded that there is an increased administrative burden on nurses in the healthcare setting besides their usual nursing care practices. Nurses are more engaged in the administrative work and have little or no time for patient care. Participants explained that nurses were wearied owing to an overload of patients, which reduced their caring ethics.

Nurses found themselves not coping well owing to the high numbers of patients versus the few nurses. The shortage of nurses resulted in overloaded and overworked nurses in the nursing unit. High levels of expectations were placed on nurses in spite of their smaller number, and this resulted in nurses having negative attitudes towards the patients and thus affecting their pledge of service concerning the aspect of caring. This category was supported by the following quotes:

‘In the public hospital, it is very sad. It is difficult to give 100% care. It is impossible. Sometimes, you have three critically ill patients in one cubicle, and other 10 patients to attend to. You still need to write the report of everyone, and while still writing the report, the other patient complicates and sometimes you are only two nurses in the cubicle. Now you are rushed to change and make patients clean, so that the family finds a clean patient.’ (Participant 1, aged 47 years, female)

‘I think there is also lack of caring among nurses, I think it is because of shortage again, they end up being burnt out. One will find that two registered nurses are working with lots of patients, and they say that they are going to do only that which they can, because there is too much work for too little nurses.’ (Participant 2, aged 37 years, female)

Nolte et al. (2017:4366) described compassion fatigue as a state of exhaustion that is reliant on a caring relationship with a loss of coping ability amongst nurses. The consequences of compassion fatigue include sleep disturbance, hypervigilance, fear, anxiety, difficulty concentrating, physical sensations such as tight muscles, feeling burdened, fatigued and overwhelmed with hopelessness and isolation, along with disengagement. MacPhee, Dahinten and Havaei (2017:3) mentioned that heavy nurse workloads were associated with burnout and emotional exhaustion. Without adequate resources and support to meet workload demands, nurses grow dissatisfied, emotionally exhausted and experience being burnt out.

Participants reported that the negative attitude of nurses towards patients affected caring. The negative attitude of nurses resulted from anger that emanated from various factors such as high number of patients, increased workload, shortage of nurses and poor remuneration. Participants also reported that nurses’ anger reduced their productivity and resulted in patients ended up being neglected, as evident in the following quotes:

‘Nurses are angry, so much anger. Most of them are overworked due to high number of patients in the unit, they are also short staffed, and they are not paid very well. Patients end up being neglected because of the anger and when nurses are angry their productivity becomes affected.’ (Participant 3, aged 25 years, female)

‘Patients tell you that nurses are rude, they shout at them. When a nurse shouts at one patient, others are also affected. This makes patients to be scared to ask nurses anything because they will be treated like the one who has been shouted at.’ (Participant 4, aged 29 years, male)

Nolte et al. (2017:4366) affirmed that there are reports of patient abuse by nurses, including hitting, slapping and neglecting of patients. Such behaviour in nursing may be a consequence of what has been described as compassion fatigue. The result of anger amongst nurses is failure to care, poor quality of care and nurses who cannot provide care during the critical times of illness. Woith et al. (2017:212) reinforced the fact that nurses’ negative attitudes and implied biases towards the patients could affect their ability to provide compassionate care to vulnerable patients, further contributing to poor health outcomes. Woith et al. (2017:212) further stated that patients reported feeling lonely; they said they were dehumanised and ignored by the nurses on a daily basis. Patients described being judged and treated with a lack of empathy and compassion; they stated their care was rushed and they were seen as a number rather than as persons.

## Discussion

The objective of this study was to explore and describe the final-year student nurses’ lived experience of caring for the patients. In this study, final-year student nurses experienced caring for patients as a therapeutic relationship with patients, as an integral part of caring, which was made easy by teamwork and team spirit. They also experienced continuous caring that promoted quality and safe nursing, as well as satisfaction amongst the nursing staff and the patients. The participants also expressed various barriers that contributed to a lack of caring as a negative experience.

A therapeutic relationship with the patients was an integral part of caring. The relationship created a caring atmosphere in the nursing unit where patients felt loved, welcomed and valued. The therapeutic relationship enhanced trust between the nurses and the patients, where the patients felt free to communicate with the nurses, which led to improved quality of caring in the unit. The relationship with the patients involved respecting, showing empathy, having open communication and loving kindness. Respect went beyond accepting the impression or attitude that people had a right to independent decision-making. Nurses in their professional relationships needed to practise with compassion and respect for dignity, worth, privacy and uniqueness of every individual, unrestricted by considerations of social or economic status, personal attributes or the nature of health problems (Hinkler & Cheever 2017:26). The positive impact of empathy in the nursing practice has been recognised and has been associated with the relief of pain and worry (Alkan 2017:61). Nurses’ ability to communicate with the patient, having time to listen and be attentively present was of great importance for the well-being of patients (Timmermann, Uhrenfeldt & Birkelund 2017:66). Coropes et al. (2016:4924) alluded that when patients get sick, the family, and their community, also gets sick. In this situation, nurses can go further in caring and need to have an open listening ear that favours the integral part of caring.

Teamwork and team spirit were the fundamentals of caring for the patients in the nursing unit. It enabled nurses to work together effectively and efficiently to meet the objectives of the unit in order to promote quality care. Teamwork involved open communication amongst nurses, which included having discussions amongst team members about the problems in the nursing unit, as well as giving each other feedback in addition to reporting on the condition of the patients. In order to ensure the effective implementation of quality care and decisions, there should be a discussion of the problem amongst the nursing personnel. Personnel input should be considered to enhance the quality of the suggested care or solution (Meyer et al. 2009:171).

Giving each other feedback in the nursing unit promotes effective two-way communication. The multidisciplinary team can discuss the patients’ progress and/or plan therapy, and the information can be obtained from the daily ward reports when feedback is received (Meyer et al. 2009:170).

Continuous caring in the nursing unit promoted quality and safe nursing, as well as satisfaction amongst the staff and the patients. It improved compliance to treatment and the understanding amongst the patients. The patients had to be given information about their condition and treatment to gain their cooperation, which made them aware of their condition, which in turn increased self-care at home. Continuous caring in the nursing unit decreased patient complaints, which also prompted the nurses to care for the patients without complaints and resentment. Nurses were required to open the channels of communication so that patients would not communicate to them through a suggestion or complaint box, but instead could express themselves directly to the nurses if there were problems. Kemp, Quan and Santana (2017:17) explained that patients who are involved in their care decisions and who are given information about their illness whilst in the hospital comply better with treatment than those who do not receive any information. Such patients understand the purpose of taking all their medications and understand their responsibility in managing their own health, resulting in decreased readmissions. Haftu et al. (2017:110) asserted that quality nursing care enhances the effectiveness of treatment. For example, patients treated by a compassionate caregiver show more understanding, thus improving compliance.

There were also various barriers that contributed to a lack of caring in the nursing unit. These barriers affected caring in the nursing unit by reducing communication in the unit, whereby patients were scared to apprise the nurses of their concerns and worries. An overload of patients, which left the nurses tired, resulted in the negative attitude of nurses towards the patients, wherein the focus of caring was on the routine tasks and not on interacting with the patients. MacPhee et al. (2017:3) mentioned that heavy nurse workloads were associated with burnout and emotional exhaustion. Negative attitude of nurses towards patients also affected caring. The negative attitude amongst nurses resulted from anger that emanated from various factors, such as high numbers of patients, increased workload, shortage of nurses and poor remuneration. Participants also reported that nurses’ anger reduced their productivity and resulted in patient neglect.

Woith et al. (2017:212) stated that patients reported feeling lonely; they said they were dehumanised and ignored by the nurses on a daily basis. Patients described being judged and treated with a lack of empathy and compassion; they stated that their care was rushed and they were seen as a number rather than as persons. Nurses focussed on the work to be done and forgot about caring of the patients. Most nurses viewed caring as a waste of time because they have a routine that needs to be followed and completed. Spending time getting to know and interact with the patient was perceived as neglecting their nursing duties. Taleghani et al. (2017:255) concurred that task-orientated nursing results in nurses paying more attention to physical care and obeying physicians’ orders. Their devotion to nursing duties affects the nurse–patient empathy. Instead of paying attention to patients’ psychological needs, nurses perform tasks and meet the patients’ physical needs. Task completion is considered more important than patients’ psychological well-being.

## Limitations of the study

During the initial phases of the research, some participants displayed uneasiness, which was managed by conducting a pre-interview to reassure and make them feel free. The study was conducted in one nursing college in Gauteng province; therefore, the findings of the study are not representative of all final-year student nurses in Gauteng. The study results are therefore contextual in nature.

## Recommendations

Based on the outcomes of this study, the following recommendations are made directed towards nursing research, nursing management, nursing education, nursing practice and policy development:

- Individualised caring should be emphasised for final-year nursing students as it promotes a therapeutic relationship.
- Pre-service or in-service training programme on teamwork should be designed by the nurse managers.
- Reinforcement of the usage of suggestion, complaint or compliment boxes by the patients and their families should be encouraged.
- Introduction, planning and implementation of wellness programmes for trained and training nurses should be practised.
- Replication of the study, which will include more nursing campuses from different provinces, employing different or similar research approaches, should be accomplished.
- Theoretical and clinical evaluation methodologies on caring should be employed throughout the nursing training period.
- Patient-centredness in caring for patients by all healthcare professionals should be introduced and promoted.
- Patient and family support systems should be formulated.

## Conclusion

The purpose of this research was to develop an understanding of final-year student nurses’ experiences of caring for patients and to formulate recommendations to facilitate caring so that these student nurses could become more caring towards their patients.

The results indicated majority of positive experiences of caring that promoted quality and safe nursing care. The negative experiences, which were barriers to caring, were also highlighted. Recommendations to facilitate caring amongst final-year student nurses were outlined. This will ensure that resources are mobilised to promote, maintain and restore caring and quality nursing care.
